# Carbon nanotube and carbon dot mediated plasmid DNA delivery in cowpea leaves

**DOI:** 10.1371/journal.pone.0340716

**Published:** 2026-01-27

**Authors:** Merve Saglam, Nikolaos Tsakirpaloglou, Aya Bridgeland, Robert Miller, Michael J. Thomson, Endang M. Septiningsih

**Affiliations:** Department of Soil and Crop Sciences, Texas A&M University, College Station, Texas, United States of America; VIT University, INDIA

## Abstract

CRISPR-Cas9 technology has been widely used as a key molecular biology tool for crop improvement. However, the advance of this technology has been hindered by host species- or genotype-dependent tissue culture protocols and poor transformation efficiencies. Recent research has shown that plasmid DNA delivered by single-walled carbon nanotubes (SWCNTs) and carbon dots (CDs) can diffuse through plant cell walls, enabling the transient expression of genetic material in plant tissues. However, such an experiment has not been performed in legumes, most of which are considered recalcitrant species for transformation. In this study, we aim to investigate the capability of a SWCNT or CD-based plasmid delivery system in expressing a target gene in cowpea (*Vigna unguiculata*) leaves via infiltration using the β-glucuronidase (*GUS*) reporter gene. Further, we aim to see the potential of SWCNTs and CDs for a CRISPR-Cas9 gene construct delivery system, with *phytoene desaturase* (*PDS*) as the target gene. Our results showed that SWCNTs and CDs can deliver the GUS reporter gene construct in the surrounding area near the site of the infiltration, which results in the temporary expression of GUS by observing the blue color in this area. Likewise, infiltration of the CRISPR-Cas9 vectors targeting the *PDS* gene for the knockout resulted in multiplex editing and large deletions within the target gene. Overall, our findings pave the way for overcoming conventional DNA delivery challenges. However, further research is needed to explore optimal germline targets for plant tissues to avoid chimerism and to allow for more efficient CRISPR-Cas9 editing resulting in heritable mutations.

## Introduction

CRISPR-Cas technology presents significant potential for revolutionizing plant biology and agriculture by enhancing functional genomics research, bolstering crop resilience against various stresses, and facilitating the rapid incorporation of desirable traits into crops [1,2]. Nevertheless, there are several obstacles hindering the widespread adoption of CRISPR technologies in plant improvement applications. These obstacles encompass challenges related to delivering CRISPR cargo, limitations associated with plant tissue and cell culture, and the absence of universally effective methods across plant species. Furthermore, our incomplete comprehension of plant genetic and metabolic networks impedes the development of plant varieties harboring desired traits. Additionally, introducing CRISPR-engineered plants to the market may encounter further hindrances in various countries due to regulatory constraints and societal attitudes toward acceptance [3–7].

Despite decades of progress in biotechnology, many plant species still pose challenges to genetic transformation and lengthy tissue culture procedures [8]. One major hurdle is the difficulty of delivering biomolecules into plant cells due to their rigid and multi-layered cell walls. Presently, there are only a few established methods for delivering biomolecules into plant cells, each with significant limitations. The most utilized approach, *Agrobacterium*-mediated delivery [9], is limited in several ways: it only works with variable efficiencies across plant species, it requires optimization in tissue culture procedures between different crops and between different genotypes within the same crop, and does not enable DNA-free and transgene-free editing [10]. Another popular method is biolistic particle delivery, or the gene gun [11], which can be used on a broader range of plant species but also has disadvantages, including: tissue damage at high bombardment pressures, problems with specimen size and positioning in the biolistic chamber, and multiple insertion copies within the genome. For transient expression of heterologous proteins in plants, viral vectors such as tobacco mosaic virus-based Geneware technology, potato virus X, and cowpea mosaic virus [12] are useful for the production of proteins at large scale. However, viral vectors are compatible with only certain plant species and have a limit on the size of the expression cassette, which confines the host plant selection and restricts the expression of large or multiple proteins at the same time. In addition, the deployment of viral vectors, even for transient expression of gene editing systems, may still be under regulatory scrutiny because of the pathogenic nature of viruses and the possible integration of viral genetic material into the plant host genome [13]. Another limitation that restricts the use of viral vectors in genome editing concerns their ability to transmit the intended mutations to future generations. However, new research and development in the nanotechnology field promises to overcome these challenges by offering various routes of transient DNA delivery through nanoparticle-mediated methods. Carbon-based nanomaterials comprise a huge group; among them, single-walled carbon nanotubes (SWCNTs) and carbon dots (CDs) have shown enormous potential in plant growth regulation, cell wall penetration, acting as delivery vectors, and biosensors [14–17].

There are extensive applications of SWCNTs in delivering genetic material. Normally having diameters from 1 to 3 nm, they give high aspect ratios and surface area-to-volume ratios. This enhances their surface chemical modifications and, therefore, DNA attachment. It also has been demonstrated that SWCNTs surface functionalization using such molecules as arginine or polymers like polyethylenimine (PEI) or chitosan increases the capacity of these nanotubes to load DNA effectively [16,18,19]. Beyond the role in delivery, SWCNTs may protect DNA from enzymatic degradation inside cells [20]. Recent studies showed that DNA was successfully delivered and the transient expression of green fluorescent protein from a plasmid was enabled by SWCNTs functionalized with PEI within intact leaf cells of diverse plant species. [19]. In these proof-of-concept studies, PEI-SWCNT complexes were used to deliver binary and non-binary plasmids containing an overexpressed GFP (mGFP) cassette into the nucleus of plant cells. The expression of GFP was seen only in cells treated with PEI-SWCNT-pDNA, while in the case of cells treated with only plasmid DNA and PEI, there was no expression. Likewise, chitosan-SWCNT complexes have been used to deliver a plasmid encoding GFP into chloroplasts. The release of DNA was favored by the alkaline pH of chloroplasts (pH 8), which resulted in the detection of GFP only in chloroplasts through fluorescence microscopy [16]. Thus, SWCNTs are considered to be good carriers for delivering DNA plasmids and making possible the transient expression of transgenes, including CRISPR-Cas9 genome editing components, in plant cells [16,19,20].

CDs are also promising drug carriers due to their small size, ability to penetrate cell membranes [21–23], low toxicity, and compatibility with biological systems [24]. They can also be easily functionalized and have been shown to effectively bind to DNA or RNA molecules for cellular delivery [25]. According to several reports [26–28]; CDs with amine groups on their surface, which results in positive charges, can bind to negatively charged DNA or RNA by electrostatic interaction to form CDs-DNA/RNA complexes and deliver the attached DNA or RNA into cells. To enhance their binding efficiency, positively charged CDs can be synthesized using precursors like polyethyleneimine (PEI) or chitosan [29]. These nanomaterials offer advantages such as high aspect ratio, biocompatibility, and fluorescence properties, enabling efficient delivery and tracking of genetic material within plant tissues [30]. Carbon dots were utilized in Wang et al.‘s study [25] to transport DNA into both rice root cells and callus. Similarly, Schwartz et al. 2020 [31] employed carbon dots to introduce small RNA molecules, inducing RNA interference (RNAi) in mature leaves of the model plants *Nicotiana benthamiana* and tomato.

Most legumes are recalcitrant, hindering the efforts to make speedy progress for crop improvement via genetic engineering or gene editing. To our knowledge, the use of nanoparticles for DNA delivery systems in legumes has not been reported yet. Therefore, our study aims to explore the suitability of CNTs and CDs as carriers for transient exogenous DNA delivery and expression in cowpea (*Vigna unguiculata*) leaves. Once optimized, further efforts can be directed to other parts of plant tissues to avoid problems with chimerism and to bypass tissue culture to quickly and efficiently perform gene editing in legumes.

## Materials and methods

### Plant materials

All the cowpea materials used in this study were from the cowpea variety IT97K-499–35 [32]. The greenhouse facility at Texas A&M University, College Station, Texas, was used for seed multiplication. The plants were grown in pots in 16/8-hour light/dark cycles and day/night temperatures of approximately 32/26°C.

### PEI-SWCNTs and PEI-CDs preparation

Polyethylenimine-functionalized single-walled carbon nanotubes (PEI-SWCNTs) were prepared as described previously [19,33]. Briefly, single-walled, carboxylic acid functionalized carbon nanotubes (Cat. no. 652490, Sigma-Aldrich, St. Louis, MO, USA) were ﬁrst covalently modiﬁed with the cationic polymer polyethylenimine (PEI, branched, molecular weight 25,000; Cat. no. 408727, Sigma-Aldrich, St. Louis, MO, USA) to carry a net positive charge in preparation for attaching plasmid DNA. Following the published protocol, zeta potential was measured by Zetasizer Nano ZS (Malvern Panalytical, Malvern, UK) and determined within the appropriate +50 to +70 mV range before continuing. Fresh PEI-CNTs were stored in aliquots at 5°C and prepared fresh every month or until agglomeration of nanoparticles became visible to the naked eye. Carbon dots (CDs) were synthesized through a modified hydrothermal method [34–36]. Briefly, 1 g of glucosamine hydrochloride was triturated and mixed with 1.35 mL of 4,7,10-trioxa-1,13-tridecanediamine (TTDDA) in 20 mL of deionized water. This mixture was microwaved at 700 W for 3 minutes, then cooled to room temperature. The synthesized CDs underwent extensive purification by repeated washing with 50 mL aliquots of chloroform (or dichloromethane) and 10-min bath sonication cycles until the supernatant became clear. After washing, the aggregated CDs were resuspended in 20 mL of deionized water and sonicated for 10 min. This suspension was then filtered using a 10,000 Da molecular weight cut-off filter via centrifugation at 4,000 rpm for 2 hours, followed by passage through a 0.2 µm syringe filter. The filtrate was collected and stored at 4°C for two days. For subsequent acid functionalization, the aqueous phase was removed using a vacuum spin dryer. The dried CDs were then resuspended in methanol at a concentration of 10 mg/mL and sonicated for 10 min. After filtration through a 0.2 µm syringe filter, succinic anhydride, at half the mass of the CD filtrate, was added, and the solution was vigorously stirred overnight. The methanol was then evaporated using a vacuum spin dryer. The acid-decorated CDs were further purified by washing with 0.5 mL of tetrahydrofuran via bath sonication until the supernatant was clear. Finally, the CDs were resuspended in 0.5 mL of methanol, dried using a spin dryer, and stored as a viscous oil. These acid-decorated CDs were functionalization with branched polyethylenimine (PEI, branched, molecular weight (MW) 25,000; Sigma-Aldrich, cat. no. 408727) following the protocol for PEI reaction with COOH-SWNTs [19]. Subsequently, plasmids were attached electrostatically, in different ratios of plasmid DNA and functionalized SWCNTs or CDs, as described previously [19,33]. Fresh PEI-SWCNTs or PEI-CDs were stored in aliquots at 4°C until agglomeration of nanoparticles became visible to the naked eye.

### Preparation of plasmid constructs

This study utilized the binary and non-binary versions of the GUSPlus (pCAMBIA 1305.1 (pr35S::GUSPlus), size: 11.8 kb; pUC19-[pr35S::GUSPlus], size: 5.4 kb) [33] and the *VgPDS* plasmids (pTRANS_100-*VgPDS*; size: 8.6 kb) [37] to attach to both SWCNTs-PEI and CDs-PEI complexes for subsequent leaf infiltration.

### Leaf Infiltration with SWCNTs-PEI-plasmid and CDs-PEI-plasmid complexes

**Fig 1:**
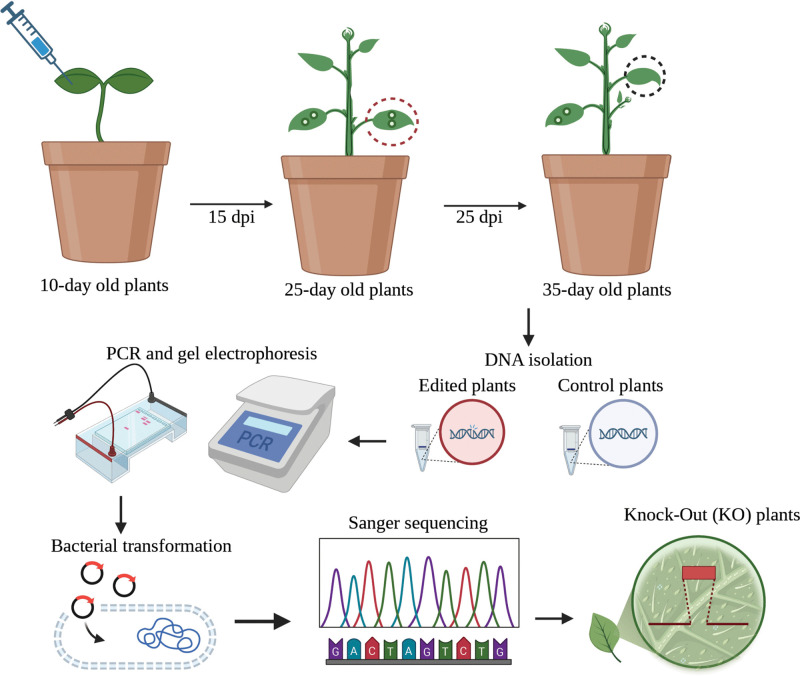
Scheme of CNT-Mediated Gene Knockout in Plants. Ten-day-old plants were infiltrated with CNTs and CDs containing CRISPR-Cas reagents, followed by sample collection at 15-days post-infiltration (dpi). Edited and control plants had their DNA isolated, PCR-amplified, and analyzed by gel electrophoresis. Sequencing confirmed the presence of mutations, and phenotypic changes served as validation for the knockout (created with BioRender.com).

To deliver the SWCNT-PEI-plasmid or CD-PEI-plasmid complexes, a small, mechanical perforation was made on the abaxial surface of leaves from 10- to 14-day-old cowpea plants (grown under greenhouse conditions) using a needle (Fig. 1). For infiltration, the complexes were prepared at mass ratios of 1:1 or 3:1 (complex:plasmid). Approximately 1 mL of the respective complex was infiltrated once into two leaves of the same plant. The plasmids encoded either the *β-glucuronidase* (*GUS*) gene cassette for reporter assays or the necessary components for genome editing. The genome-editing plasmids comprised the Cas9 cassette and single guide RNAs (sgRNAs) designed for multiplex editing of the *VgPDS* gene, as previously established [37]. Subsequently, the plants with infiltrated leaves were kept at greenhouse conditions for 72 hours post-infiltration (GUS assay) or until an albino phenotype was observed (Cas9-*VgPDS*). As previously described, GUS enzymatic activity was visualized by histochemical assay [38]. Leaves infiltrated with MES buffer, SWCNTs, CDs, or plasmid DNA solutions were used as negative controls. GUS expression and the albino phenotype were documented using an Olympus SZX10 microscope under brightfield settings with exposure time set at 100 ms.

### *VgPDS* Knockout and sequencing analysis

Genomic DNA was isolated from infiltrated leaves as previously described [39]. The target region was then amplified using Phusion High-Fidelity DNA polymerase (Thermo Fisher Scientific) and primers overlapping the sgRNAs region of the *VgPDS* gene. To identify edits in the *PDS* gene, PCR amplification was performed using primers overlapping the sgRNA region of the gene (F1: 5’-TGCATGTTTTTAATTCAGGCGT-3’ and R1: 5’-CCAGTCTCCATCCTCATCTTTC-3’). The resulting PCR products were analyzed by 1% (w/v) agarose electrophoresis, and the corresponding amplicons were excised from the gel using the Qiagen QIAquick Gel Extraction Kit. Subsequently, the extracted amplicons were cloned in Escherichia coli (Dh5a strain) using the Zero Blunt TOPO PCR Cloning Kit (Thermo Fisher Scientific) and 5 clones per amplicon were submitted for Sanger Sequencing (Eurofins Genomics). Sequencing results were analyzed by mapping them to the unedited sequence using the MAFFT Multiple Sequence Alignment Software Version 7 [40] within Benchling (www.benchling.com).

## Results

### Transient GUS expression after CNT- or CD-mediated plasmid delivery

**Fig 2:**
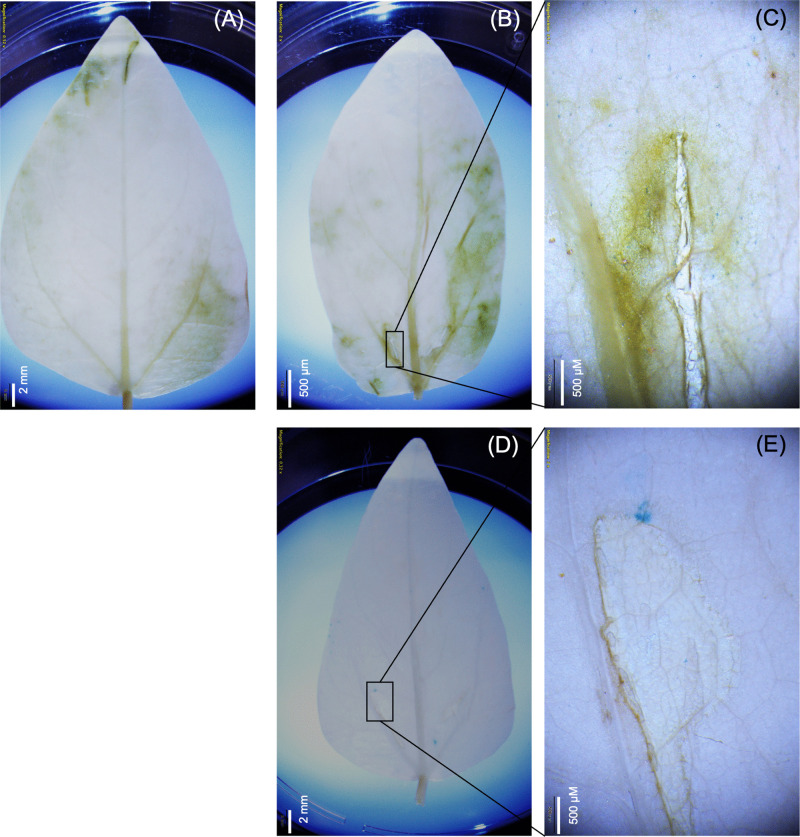
Histochemical detection of GUS expression in cowpea leaves infiltrated with carbon nanotubes (CNTs) and plasmid DNA (pDNA) solution. Cowpea leaves were treated with a mixture of CNTs and pDNA at a 1:1 ratio (500 ng of CNTs to 500 ng of pDNA). After a 72-hour incubation period, GUSPlus enzymatic activity was assessed using a histochemical assay following standard procedures. Panel (A) shows cowpea leaves infiltrated with water as the negative control. GUS expression was observed in cowpea leaves infiltrated with the CNT-pDNA mixture, utilizing both binary (Panels B-C) and non-binary plasmids (Panels D-E). The exposure time was set to 100 ms.

**Fig 3:**
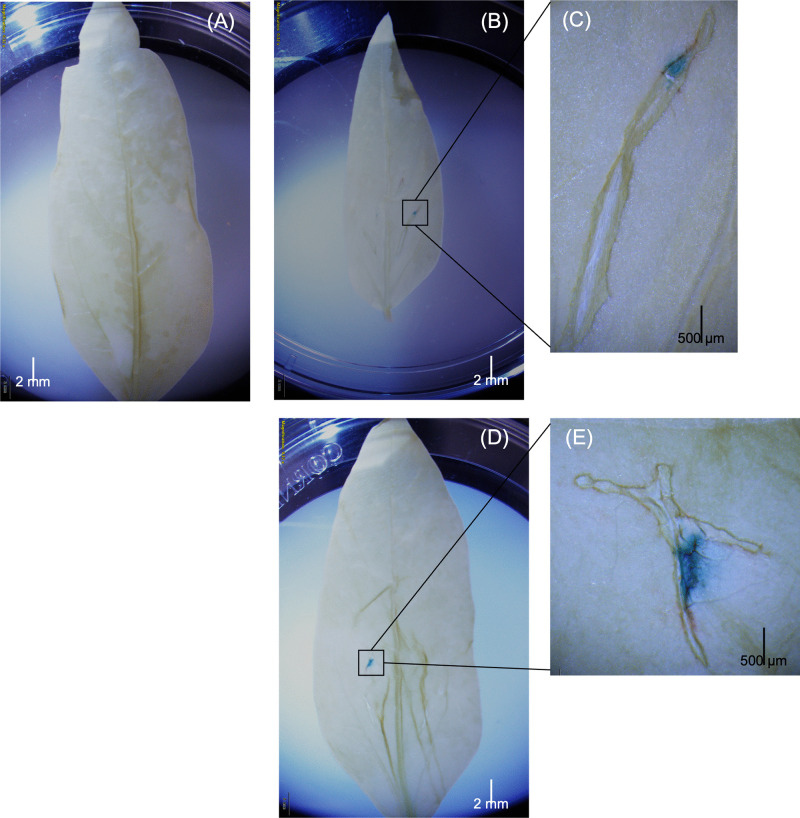
Histochemical detection of GUS expression in cowpea leaves infiltrated with carbon dots (CDs) and plasmid DNA (pDNA) solution. Cowpea leaves were treated with a mixture of CDs and pDNA at a 1:1 ratio (500 ng CDs: 500 ng pDNA). After a 72-hour incubation period, GUSPlus enzymatic activity was assessed using a histochemical assay according to standard procedures. Panel (A) shows cowpea leaves infiltrated with water as a negative control. GUS expression was observed in cowpea leaves infiltrated with the CNT-pDNA mixture, using both binary (B-C) and non-binary plasmids **(D-E)**. The exposure time was set to 100 ms.

A GUSPlus assay was performed to evaluate the effective delivery and expression of CNT-pDNA and CD-pDNA constructs in cowpea leaves. Both binary and non-binary vectors encoding the GUSPlus enzyme were employed to assess transient expressions mediated by carbon nanotubes (CNTs) and carbon dots (CDs). The leaves were infiltrated with solutions of CNT-pDNA or CD-pDNA and allowed to rest for three days to facilitate the expression and accumulation of GUSPlus protein. After formaldehyde fixation, the chlorophyll was cleared from the leaves, followed by histochemical staining. A blue coloration, which indicates successful GUSPlus expression, was observed in both binary and non-binary plasmids and in both delivery systems, exclusively in the GUSPlus-treated leaves (Figs 2 and 3). In contrast, control leaves remained colorless. GUSPlus activity was particularly concentrated at the infiltrated sites, and it extended along the vein pathways. In addition to the 1:1 ratio, we also tested a 3:1 nanoparticle-to-plasmid mass ratio for both CNT and CD treatments as part of our initial evaluation, and the 1:1 ratio was determined to be superior. Representative results are provided in the Supplementary Information for the 3:1 ratio for CNTs (S1 Fig) and CDs (S2 Fig).

### Testing CNT and CD delivery of CRISPR-Cas for gene editing of cowpea leaves

**Fig 4:**
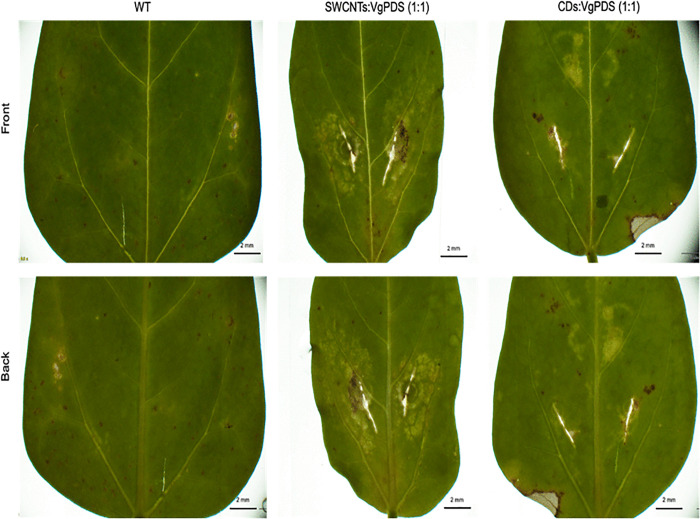
Phenotypes of cowpea leaves infiltrated with single-walled carbon nanotube (SWCNT) or carbon dot (CD) plasmid vector mixtures. Cowpea leaves were infiltrated with a 1:1 mixture of SWCNTs or CDs and *VgPDS* plasmid DNA solution (a non-binary vector) at a concentration of 500 ng each. After 10 days of incubation, white spots appeared on both the front and back sides of the infiltrated leaves. Genomic DNA was then extracted from these leaves for PCR screening, while non-infiltrated leaves served as negative controls. The exposure time for the infiltration was 100 ms.

We focused on transient gene editing in cowpea leaves to test whether carbon nanotubes (CNTs) or carbon dots (CDs) can effectively deliver CRISPR-Cas reagents into plant tissues. We infiltrated the cowpea leaves with mixtures of CNTs, or CDs combined with a non-binary version of a plasmid that contains the genome editing components targeting the *VgPDS* gene. After a 10-day incubation period, we observed the characteristic albino phenotype, which indicates a knockout of the *VgPDS* gene, around the infiltration sites for both types of complexes (see Fig 4). Following this, we extracted genomic DNA from the entire leaf for molecular screening of the target region to verify the disruption of the *VgPDS* gene’s function. We also included non-infiltrated leaves and leaves infiltrated with MES buffer or water as negative controls. In total, we performed two infiltration points on each of the four leaves from four separate plants.

### DNA sequencing of phenotypically impaired leaves

**Fig 5:**
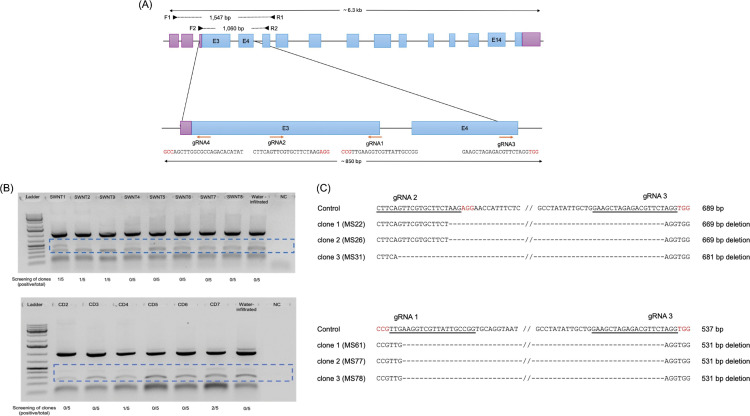
Mutations of the PDS target region identified from cowpea leaves infiltrated with SWCNT-PEI-VgPDS or CD-PEI-VgPDS plasmid vector mixtures. **(A)** Schematic illustration of the PDS gene (VgPDS; Vigun01g249800) structure in cowpea, highlighting the translated regions and untranslated regions (UTRs). Translated exons are marked in blue, while UTRs are shown in grey. The positions of four selected guide RNA (gRNA) target sites are indicated by orange arrows for the multiplex editing of *VgPDS* gene, with each gRNA sequence provided and protospacer adjacent motif (PAM) sequences underlined. Primer sites (F1 and R1) used to detect deletions within the targeted region are represented by arrowheads. The schematic includes the lengths of the entire gene, the targeted region, and the expected PCR amplicon from the wild-type locus. **(B)** Agarose gel electrophoresis analysis of the *VgPDS* target region amplified with the F2 and R2 primers from genomic DNA extracted from cowpea leaves (wild type (WT) allele amplicon size: 1,062 bp) infiltrated with SWCNT-PEI-VgPDS or CD-PEI-VgPDS plasmid vector mixtures. The boxed region in the gel image represents the excised fragment subsequently recovered from the gel. The purified DNA was cloned into TOPO vectors, and five plasmid clones per sample were sequenced by Sanger sequencing. The number of plasmids with identified deletions per sample is indicated below the respective lanes in the gel image. The negative clones had unedited sequences similar to the WT allele of *VgPDS* gene. L: Thermo Scientific GeneRuler 1 kb Plus DNA Ladder (Thermo Fisher Scientific, Cat. No. SM1331). NC: negative control. **(C)** Alignment of DNA sequences from deletion events identified using different primer sets on DNA extracted from leaves infiltrated with either SWCNTs-PEI-plasmid or CDs-PEI-plasmid vectors targeting the PDS gene, which displayed observable phenotypes. The reference sequence of the unmodified VgPDS locus is displayed at the top, followed by sequences of three representative clones showing deletions. The corresponding leaf for the origin of the amplicon/clone is also indicated in brackets. The sizes of the control site and each deletion are provided. Targeted gRNA sequences are underlined, and PAM sequences are highlighted in red.

A schematic of the cowpea phytoene desaturase (*PDS*) gene (*VgPDS*; Vigun01g249800) was generated to illustrate its structural features and the specific sites targeted for CRISPR-Cas9 editing (Fig 5A). In this diagram, translated exons are represented in blue, while untranslated regions (UTRs) are shown in gray. Four guide RNA (gRNA) target sites within the *VgPDS* gene are marked by orange arrows, with the nucleotide sequences of each gRNA displayed near their target sites. To validate successful editing, genomic DNA was extracted from cowpea leaves infiltrated with SWCNT-PEI-VgPDS or CD-PEI-VgPDS plasmid mixtures and amplified using primers F2 and R2 (Fig 5B). PCR products were separated by agarose gel electrophoresis, and the specific region of the gel containing the target amplicons was excised for further analysis. DNA was recovered from the gel, cloned into TOPO vectors, and Sanger sequenced to confirm deletions within the target region (Fig 5C). For each sample, five plasmid clones were sequenced. For the SWCNT delivery system, deletions were identified in 3 out of 40 plasmid clones (7.5%). These deletions ranged from 669 bp (2 clones) to 681 bp (1 clone) within the sgRNA2-sgRNA3 region and were found in samples from three separate infiltrated leaves. Similarly, for the CD delivery system, 3 out of 30 plasmid clones (10%) showed deletions. All deletions observed with CD delivery were 531 bp in length, occurring between the sgRNA1 and sgRNA3 sites; two of these clones were derived from the same leaf.

## Discussion

The advancement of plant genetic engineering technology holds great promise for addressing the decreasing supply of food and energy resulting from climate change and the demographic and socioeconomic issues facing humanity. Even with the numerous advancements in plant gene editing technologies that have been made, there remains room for improvement concerning the means of delivering and the expression of genome-editing components to plants intracellularly, particularly in intact plants [41]. Consequently, to achieve pDNA transfection and expression, a nanomaterial-based delivery system can be used that inserts pDNA into the plant cell nucleus in a way like a nucleosome while enabling the expression of the attached biomolecule. This nanomaterial accomplishes exogenous DNA transfection and expression without causing toxicity or tissue damage to the plant, nor integrating into the plant genome [19]. Previous studies have been shown that polymer-functionalized SWCNTs, at working concentrations used for gene delivery and expression studies (<10 mg L ⁻ ¹), do not induce cytotoxicity or tissue damage in plants. In our study, we used 500 ng of CNTs, or CDs diluted in 500 µL of MES delivery buffer, corresponding to a final concentration of 1 mg L ⁻ ¹, which remains well below the reported non-toxic threshold [16,19].

CNTs and CDs for plasmid DNA delivery in cowpea leaves represent a pivotal advancement in plant genetic engineering. The emergence of nanotechnology opens up new avenues for the efficient gene delivery systems required for the enhancement of crop traits, thus responding to the challenge of agricultural productivity. Among the carriers, CNTs and CDs are considered to be the most promising due to their unique properties of high surface area, biocompatibility, and facilitating the transport of nucleic acids across cellular barriers, and the transient expression of the biomolecules attached to them [42]. Recent studies have highlighted the potential of using carbon nanotubes (CNTs) and carbon dots (CDs) loaded with plasmid DNA to penetrate plant cell walls, although these techniques are yet to be confirmed in legume species [43,44].

So far, the efficiency of CNTs as a carrier of plasmid DNA insertion and expression into plant cells has been documented in several plant species. Single and multiple-wall nanotubes (SWNTs and MWNTs) have been used as plant gene vectors. Demirer et al. 2019 [19] explored using positively charged materials like PEI, chitosan, and sodium dodecyl sulfate (SDS) to modify SWNTs and MWNTs for nucleic acid delivery. Decorated SWNTs have successfully expressed GFP and YFP in *Nicotiana benthamiana* leaves and even chloroplasts. Previously, our lab also demonstrated that PEI-functionalized CNTs can successfully deliver plasmid DNA into rice leaf and embryo tissues [33]. Pinyokham et al. showed that CNTs could deliver plasmid DNA into rice calli with efficiency and hence could transiently express the gene in plant cells [42]. This aligns with findings from Wang et al., who noted that CNTs could mediate genetic engineering in plants, emphasizing their role in overcoming limitations of traditional methods such as Agrobacterium-mediated transformation [45]. CNTs’ ability to penetrate rigid plant cell walls is particularly relevant for species like cowpea, where conventional methods often face challenges.

Additionally, CDs have gained attention as effective nanocarriers for plasmid DNA. Their small size allows them to evade size exclusion limits imposed by plant cell walls, enhancing their potential for effective gene delivery. Wang et al. highlighted that CDs could facilitate the delivery of functional DNA in plants, further broadening their scope in plant genetic engineering [25]. Furthermore, Thakur’s research on chitosan-PEI passivated carbon dots demonstrated effective DNA binding, which is essential for delivering intact plasmid DNA into target cells [46].

Electrostatic interactions are employed for both CNTs and CDs during their interactions with plasmid DNA to form stable nanocomplexes, which ensure that successful cellular uptake and the eventual expression of genetic material take place. Also, the delivered nanomaterials interact more significantly when targeted at organelles like chloroplasts for enhancing gene expression. In fact, Santana et al., using targeted carbon nanostructure delivery of genetic materials, demonstrated high enhancement of photoynthetic efficiency and enhanced crop yields [47].

In our experiments, varying ratios of plasmid DNA to CNTs were tested to determine the optimal conditions for leaf infiltration. We tested 1:1 and 3:1 nanoparticle-plasmid ratios during the infiltration experiments, as part of our initial delivery optimization. The inclusion of both ratios was intentional and based on prior reports. These ratios were used to preliminarily assess the delivery efficiency for both CNT and CD formulations. Our results showed that the 1:1 ratio yielded the highest fluorescence, consistent with findings by Demirer et al. 2019 [19]. However, the efficiency of CNT-mediated delivery can depend on plasmid size, promoter type, and plant species. For example, it was able to deliver large binary plasmids as well as smaller non-binary plasmids; through GUS imaging, the signals were stronger compared to the negative controls. Though smaller plasmids were expected to perform better because of easier cellular uptake, no remarkable differences in fluorescence were seen when driven by the same promoter.

One advantage offered by the ability of CNTs to penetrate cell walls and membranes in plants without tissue damage, in contrast to other physical/chemical transfection methodologies (electroporation), as discussed by Tonelli et al. 2015 [48] and Cunningham, et al. 2018 [49], involves enhanced cell proliferation, which is indicative of high regeneration of transformed tissues through the action of CNTs, as has also been shown by Khodakovskaya et al. 2012 [50]. These properties make CNTs a valuable tool both for transient and stable gene expression studies. Furthermore, integration of carbon nanomaterials with other systems, such as lipid nanoparticles, may enhance the efficiency of plasmid DNA delivery. In this context, Zhu et al. showed that the combination of nanocarrier systems could prolong gene expression and thus indicated possible synergy between different delivery platforms [51].

In future optimization, several aspects have to be studied, such as functionalization of CNTs and CDs for better interaction with the plant cell, refinement in the vacuum infiltration protocol for successful transfection, and other targeted delivery approaches to route nanoparticles into specific cellular organelles like chloroplasts. Further experimentation is also required to target germline tissues and assess the inheritance of target mutations in the progeny to determine the stability of the genetic modifications.

The implementation of CNTs and CDs as vectors in plasmid DNA delivery and expression in cowpea leaves demonstrates the usefulness of this approach in legumes. Their unique properties, their cultivar/species-independent transformation, and their potential ability to overcome regeneration bottlenecks in tissue culture by enabling in planta transformation provide a sound platform for non-transgenic gene editing. While remaining challenges exist, further research and optimization studies will pave the way towards more innovative solutions for agricultural biotechnology.

## Supporting information

S1 Fig Histochemical detection of GUS expression in cowpea leaves infiltrated with CNTs and plasmid DNA (pDNA) solution at a 3:1 ratio (500 ng CNT: 167 ng pDNA). (PDF)

S2 Fig Histochemical detection of GUS expression in cowpea leaves infiltrated with CDs and plasmid DNA (pDNA) solution at a 3:1 ratio (500 ng CD: 167 ng pDNA). (PDF)

S1 File Raw images. (PDF)
