# Supplementary material for: Carbon nanotube and carbon dot mediated plasmid DNA delivery in cowpea leaves
Source: PLoS One. 2026 Jan 27;21(1):e0340716. doi: 10.1371/journal.pone.0340716 (PMC12843543; doi:10.1371/journal.pone.0340716)
Supplement: S1 Fig — (PDF) [file pone.0340716.s001.pdf]

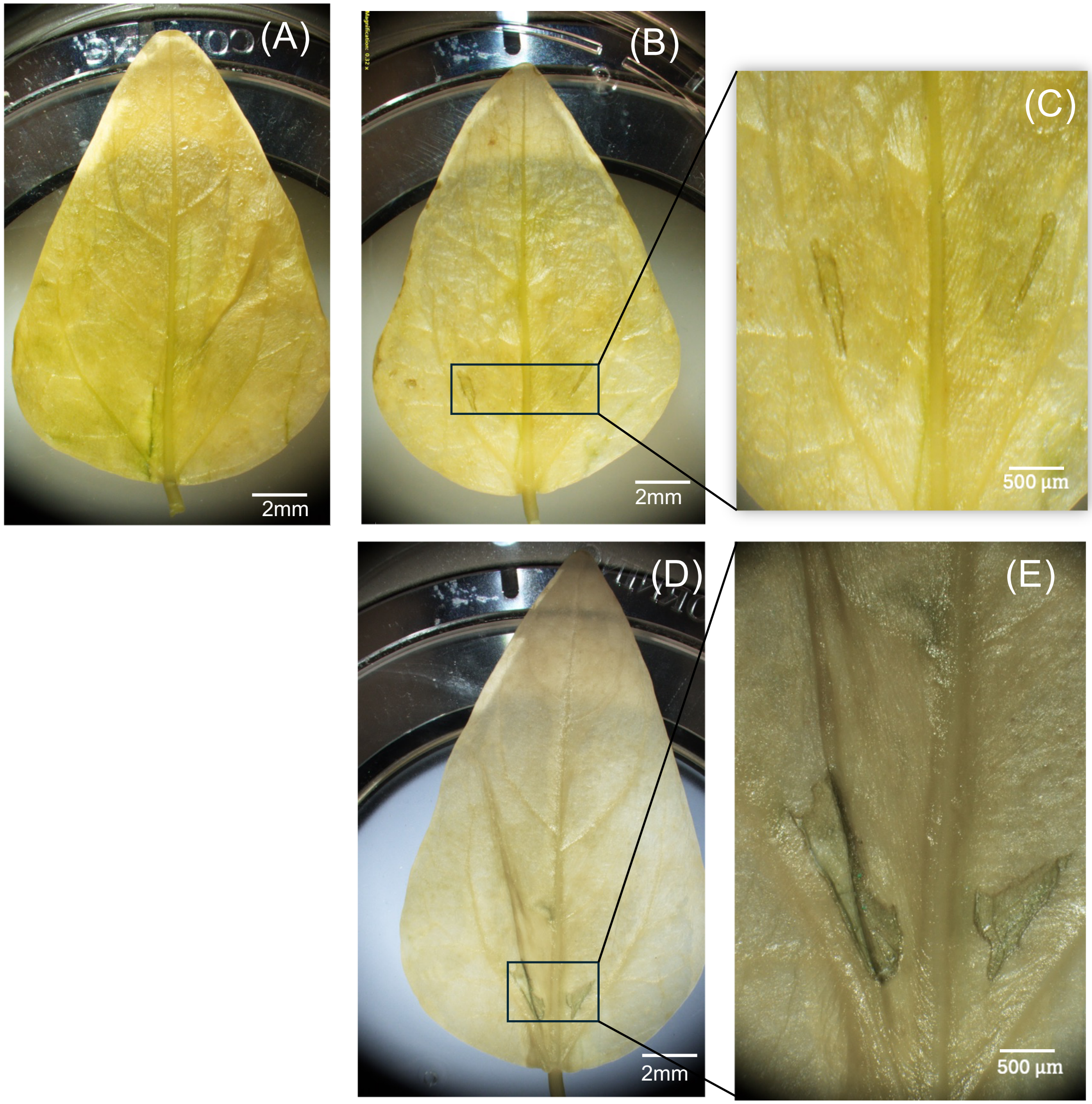

S1 Fig. Histochemical detection of GUS expression in cowpea leaves infiltrated with CNTs and plasmid DNA (pDNA) solution at a 3:1 ratio (500 ng CNT: 167 ng pDNA). After a 72 hours incubation period, GUSPlus enzymatic activity was detected using a histochemical assay following standard procedures. (A) Cowpea leaf infiltrated with water (negative control). GUS expression detected in cowpea leaves infiltrated with the CNT-pDNA mixture, using both binary (B-C) and non-binary plasmids (D-E). Exposure was set at 100 ms.
