# Supplementary material for: Carbon nanotube and carbon dot mediated plasmid DNA delivery in cowpea leaves
Source: PLoS One. 2026 Jan 27;21(1):e0340716. doi: 10.1371/journal.pone.0340716 (PMC12843543; doi:10.1371/journal.pone.0340716)
Supplement: S1 File — (PDF) [file pone.0340716.s003.pdf]

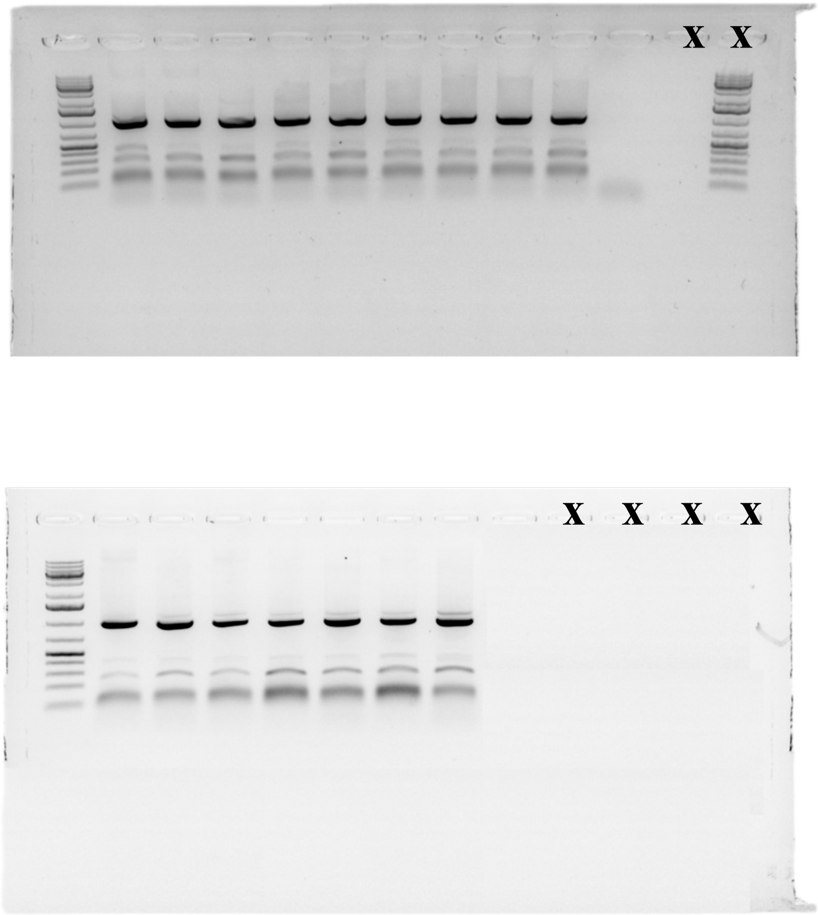

S1 Raw images. Raw agarose gel electrophoresis images of the VgPDS target region amplified with the F2 and R2 primers from genomic DNA extracted from cowpea leaves (wild type (WT) allele amplicon size: 1,062 bp) infiltrated with SWCNT-PEI-VgPDS (upper panel) or CD-PEI-VgPDS (lower panel) plasmid vector mixtures. Images were captured using Azure C200 gel documentation system. 1st lane = ladder (L): Thermo Scientific GeneRuler 1 kb Plus DNA Ladder, followed by 8 samples (upper panel) and 6 samples (lower panel), water-infiltrated control, and then negative control (NC), as labelled in Figure 5 (B).

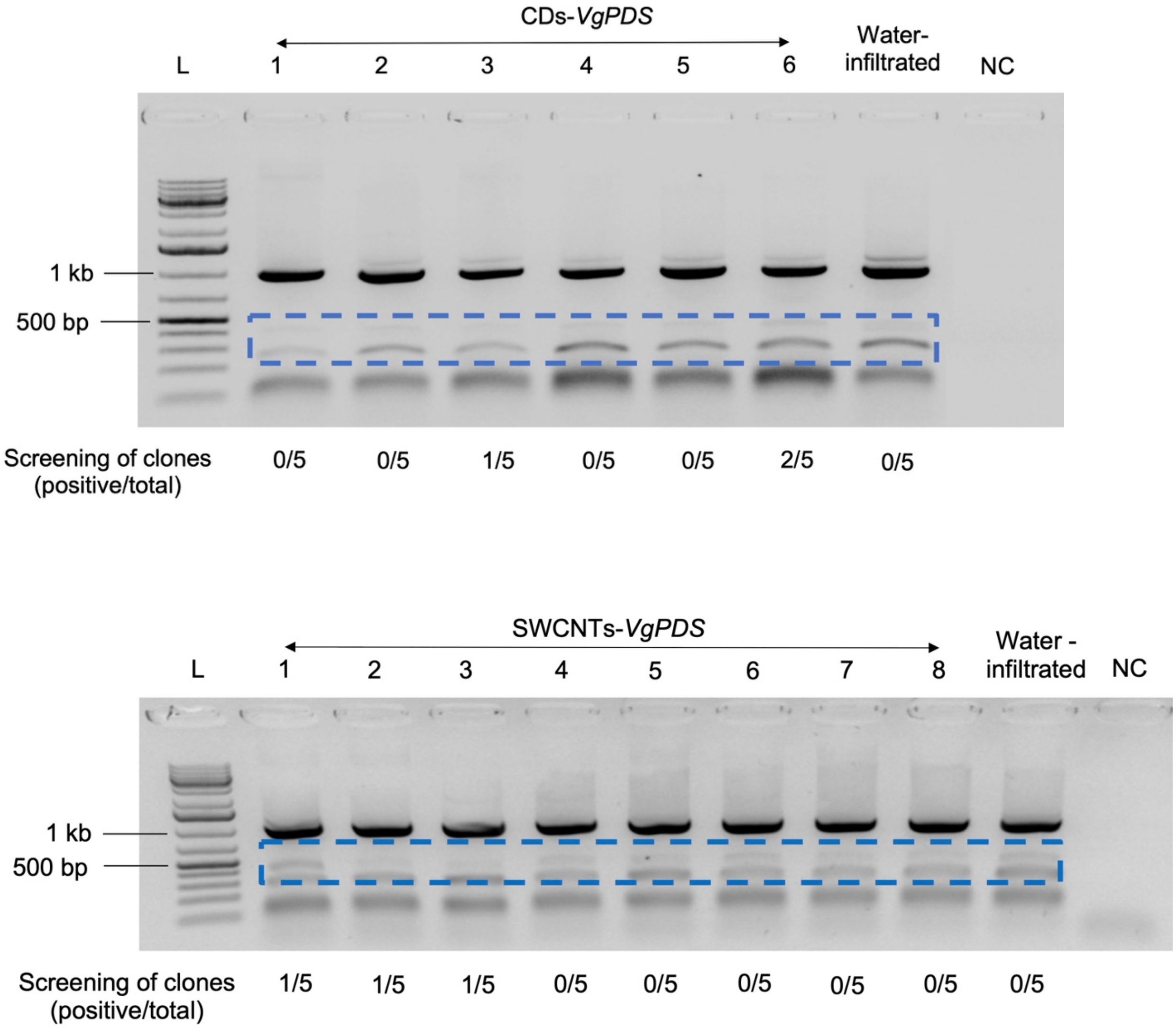

Fig 5 (B) Agarose gel electrophoresis analysis of the *VgPDS* target region amplified with the F2 and R2 primers from genomic DNA extracted from cowpea leaves (wild type (WT) allele amplicon size: 1,062 bp) infiltrated with SWCNT-PEI-VgPDS or CD-PEI-VgPDS plasmid vector mixtures. The boxed region in the gel image represents the excised fragment subsequently recovered from the gel. The purified DNA was cloned into TOPO vectors, and five plasmid clones per sample were sequenced by Sanger sequencing. The number of plasmids with identified deletions per sample is indicated below the respective lanes in the gel image. The negative clones had unedited sequences similar to the WT allele of *VgPDS* gene. L: Thermo Scientific GeneRuler 1 kb Plus DNA Ladder (Thermo Fisher Scientific, Cat. No. SM1331). NC: negative control.
